# Barium sulfate and pigment admixture for photoacoustic and x-ray contrast imaging of the gut

**DOI:** 10.1117/1.JBO.28.8.082803

**Published:** 2023-02-10

**Authors:** Hailey I. Kilian, Huijuan Zhang, Mohammad Mahdi Shiraz Bhurwani, Anoop M. Nilam, Daewoon Seong, Mansik Jeon, Ciprian N. Ionita, Jun Xia, Jonathan F. Lovell

**Affiliations:** a University at Buffalo, State University of New York, Department of Biomedical Engineering, Buffalo, New York, United States; b Canon Stroke and Vascular Research Center, Buffalo, New York, United States; c Kyungpook National University, College of IT Engineering, School of Electronic and Electrical Engineering, Daegu, Republic of Korea

**Keywords:** x-ray, photoacoustic, contrast agent, gastrointestinal imaging, barium, second near infrared window

## Abstract

**Significance:**

X-ray imaging is frequently used for gastrointestinal imaging. Photoacoustic imaging (PAI) of the gastrointestinal tract is an emerging approach that has been demonstrated for preclinical imaging of small animals. A contrast agent active in both modalities could be useful for imaging applications.

**Aim:**

We aimed to develop a dual-modality contrast agent comprising an admixture of barium sulfate with pigments that absorb light in the second near-infrared region (NIR-II), for preclinical imaging with both x-ray and PAI modalities.

**Approach:**

Eleven different NIR-II dyes were evaluated after admixture with a 40% w/v barium sulfate mixture. The resulting NIR-II absorption in the soluble fraction and in the total mixture was characterized. Proof-of-principle imaging studies in mice were carried out.

**Results:**

Pigments that produced more uniform suspensions were assessed further for photoacoustic contrast signal at a wavelength of 1064 nm that corresponds to the output of the Nd:YAG laser used. Phantom imaging studies demonstrated that the pigment-barium sulfate mixture generated imaging contrast in both x-ray and PAI modalities. The optimal pigment selected for further study was a cyanine tetrafluoroborate salt. *Ex-vivo* and whole-body mouse imaging demonstrated that photoacoustic and x-ray contrast signals co-localized in the intestines for both imaging modalities.

**Conclusion:**

These data demonstrate that commercially-available NIR-II pigments can simply be admixed with barium sulfate to generate a dual-modality contrast agent appropriate for small animal gastrointestinal imaging.

## 1 Introduction

Multimodal imaging contrast agents have been developed for a range of biomedical applications.^1^ The combination of photoacoustic imaging (PAI) with x-ray computed tomography (CT) has been explored in prior preclinical studies using functional nanoparticles.^2^^,^^3^ PAI is a low-cost imaging modality that can detect optical contrast at depth without ionizing radiation,^4^^–^^11^ while x-ray and CT contrast imaging are powerful whole-body imaging techniques used in clinical diagnostic imaging.^12^^–^^14^ In PAI, optical contrast agents that absorb in the near infrared region-II (NIR-II) have emerged as promising since they can be imaged at deeper penetration depths with reduced tissue scattering in the 1000 to 1700-nm region.^4^^,^^15^^–^^21^ Small molecule chromophores with defined molecular structures have proven to be useful for PAI in the NIR-II.^22^^–^^25^ Our group has recently demonstrated that commercially available NIR-II dyes can be formulated with biocompatible surfactants for parenteral administration to mice.^26^^,^^27^

Numerous orally ingested contrast agents have been developed, with perhaps none being as commonly used as barium sulfate.^28^ Barium sulfate is a generally safe and inert material that does not interact with the human body and has commonly been used in CT imaging of the gastrointestinal tract to provide a method for visualizing transit.^29^^–^^31^ Another common application is its usage in human swallow tests. In practice, contrast agents that are used should be able to pass through the gastrointestinal tract without being absorbed into the body or degrading due to the pH environment. Absorption into the body would cause several issues including potential toxicity, disruption of background signal, and a less intense signal from the gastrointestinal tract in focus.^32^

PAI has been used in preclinical studies to image the gastrointestinal tract.^33^^–^^35^ A pilot study in healthy volunteers showed that roasted barley could be detected in the esophagus during human swallowing.^36^ NIR-II contrast agents that are compatible with the Nd-YAG 1064-nm laser wavelength often used in PAI have high sensitivity; however, these could benefit from additional imaging information. The use of a second imaging modality such as CT could be used for validation, orientation, and acquisition of signal independent of PAI.^37^^–^^39^ In combination, the use of ionizing beam energy could be minimized to only be used intermittently for orientation along with the continuous use of the non-ionizing PAI technique. A strong contrast agent for PAI would be helpful to overcome the high background scatter and absorbance of tissues, including absorbance from blood vessels, which can be detected with no contrast agent.^40^^,^^41^ PAI for guided surgeries has been used in mice with dyes and nanoparticles that absorb in the NIR-II region (1000 to 1700 nm).^42^^,^^43^

In the current study, we examined the admixture of common NIR-II dyes and barium sulfate to generate a simple to prepare dual modality orally administered contrast agent for CT and PAI.

## 2 Results

**Fig. 1:**
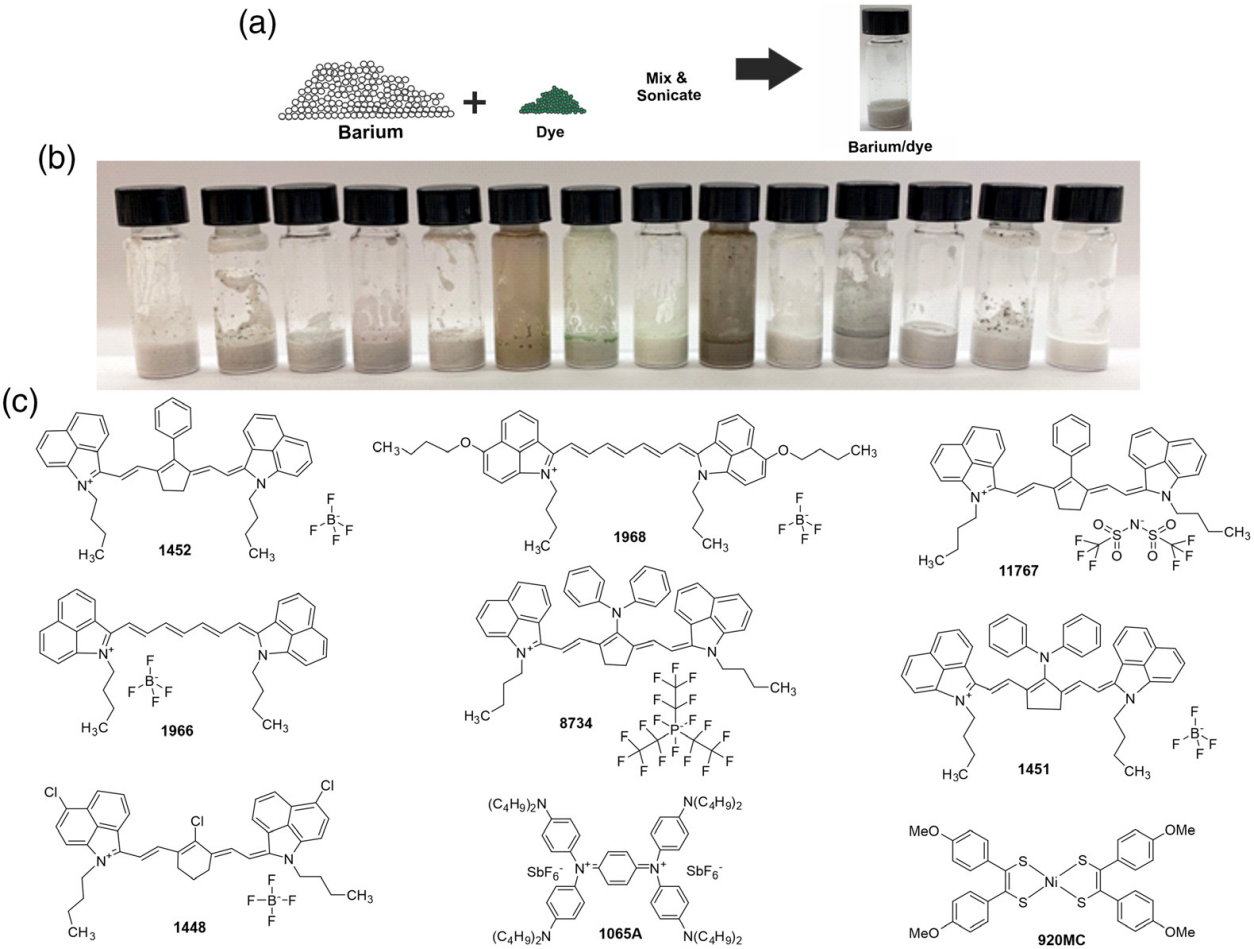
Simple generation of barium-pigment admixtures. (a) Schematic of simple formulation by admixture. (b) Left to right: 1065A, 920MC, 1451, 1452, 7630, 3958, 3734, 1906, 1448, 8734, 1966, 1968, 11767, barium sulfate alone. Samples contain 40% barium sulfate (weight by volume; w/v) in 1 ml of water with 5 mg/ml of pigment. (c) Chemical structures of some of the pigments assessed.

A mixture of barium sulfate and commercial NIR-II dyes was sonicated in water to form a simple suspension. At the concentrations used, no obvious interactions between the insoluble barium particles and the dyes were observed by visual inspection. An illustration of the formulation method is shown [Fig. 1(a)] along with a white light image of each of the thirteen dye formulations tested for a photoacoustic signal [Fig. 1(b)]. Out of the commercial thirteen dyes tested, only nine had reported structures, which are shown in Fig. 1(c). Some dyes had similar chemical pigment structures, but with different counter ions.

The contrast admixture was evaluated in an initial screening step based on three criteria: pigment separation, stickiness to the tube wall, and NIR-II absorption features. Separation of pigment and barium could lead to variation in administered doses, and would therefore be problematic; thus, relative homogeneity and minimal dye separation were desirable. Some pigments that did not become evenly suspended in the barium sulfate ended up floating on the top surface: 1065A, 920MC, and 1966. Likewise, wall stickiness was used to eliminate pigments that apparently had affinity to the surface of the glass vials that could also lead to a lack of homogeneity. 3958, 3734, 1448, and 11,767 were found to adsorb the most to the wall. The maximum absorption wavelength of each pigment, when dissolved in water or methanol, was determined experimentally and is given in Table 1. Most dyes had absorption wavelength maxima close to 1000 nm.

**Table 1:** Barium pigment admixture features.

Dye	Dye separation	Wall stickiness	$\lambda_{\max}$ in H_2_O (nm)	$\lambda_{\max}$ in methanol (nm)
1968	0	0	954	1034
920MC	2	1	1004	1096
1451	0	0	976	998
1452	1	1	1020	1024
7630	1	1	950	1066
3958	1	2	962	962
3734	1	2	896	1028
1906	1	1	964	986
1448	0	2	998	1042
8734	0	0	962	1000
1966	2	1	974	974
1065A	2	1	1034	1074
11767	1	2	1026	1024

Parameters were defined as: 0- low, 1- medium, and 2-high.

**Fig. 2:**
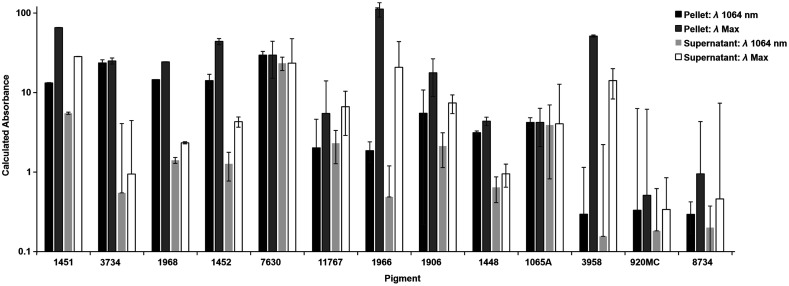
Calculated absorbances. Supernatant and pellet absorbance; depicts how much is dissolved in water and leftover in the pellet for 5 mg/ml. Samples were diluted and measured in methanol, and multiplied by the dilution factor to obtain values. Graph show mean +/− standard deviation for n=5 separately prepared samples.

Next, the NIR-II contrast parameters were determined (Fig. 2), and optical absorbance measurements were measured after centrifugation. Admixtures of each pigment were prepared, followed by centrifugation and assessing the absorbance of each at the wavelength of maximum absorption intensity and also 1064 nm. From these measurements, the calculated absorbance was determined by multiplying the measured absorbance values by the dilution factor. The absorbance measurements of the aqueous supernatant (diluted in methanol) were compared to the absorbance measurements of the pellet (re-dissolved in methanol). A dye with a high pellet and low supernatant absorbance would indicate the dye has low solubility in the mixture. Dyes with high solubility in water (1451, 7630, 1966, and 3958) were deemed to be nondesirable, since those could be more likely to get absorbed from the GI tract into systemic circulation. It should be noted that poorly water soluble dyes are not necessarily safe for oral administration since they could still be absorbed into systemic administration or alternatively become lodged in the GI tract. Nevertheless, dyes with a high pellet signal and low water soluble absorbance were advanced for further consideration. After these screening steps, the dyes that were examined further were 11767, 1452, and 1968.

**Fig. 3:**
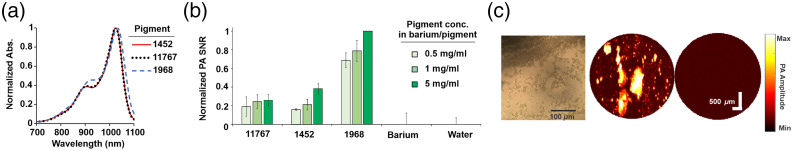
Photoacoustic features of NIR-II pigment candidates. (a) Normalized absorption spectra of indicated pigments. (b) Normalized SNR, relative to 5 mg/ml 1968 dye, of the three barium pigment admixture properties at indicated pigment concentrations. Graph shows mean +/− std. dev. from n=3 separate samples. (c) White-light micrograph of barium-1968 admixture (left). Separately prepared photoacoustic micrograph of barium-1968 (middle) or barium alone (right) demonstrating the aggregated nature of pigment in the admixture.

The three promising pigments had similar optical absorption spectra with peak absorption in methanol above 1000 nm [Fig. 3(a)]. 11,767 and 1452 correspond to the same chromophore molecular structure, just complexed with a different counterion; therefore, their identical absorption spectra are not unexpected. Phantom imaging was used to compare barium-pigment mixtures of three promising pigments [Fig. 3(b)]. Signal-to-noise ratio (SNR) results were normalized with respect to the 1968 dye at a concentration of 5 mg/ml. The signal intensity did not show a linear trend, as concentrations of 0.5 to 1 mg/ml pigment had similar SNRs. However, at 5 mg/ml pigment, PA signals increased, and hence we used the 5 mg/ml pigment concentration for further study. PAI microscopy of this formulation revealed that the dye forms relatively large microaggregates, which are larger than the barium suspension particles and do not represent a homogenous mixture [Fig. 3(c)]. 

**Fig. 4:**
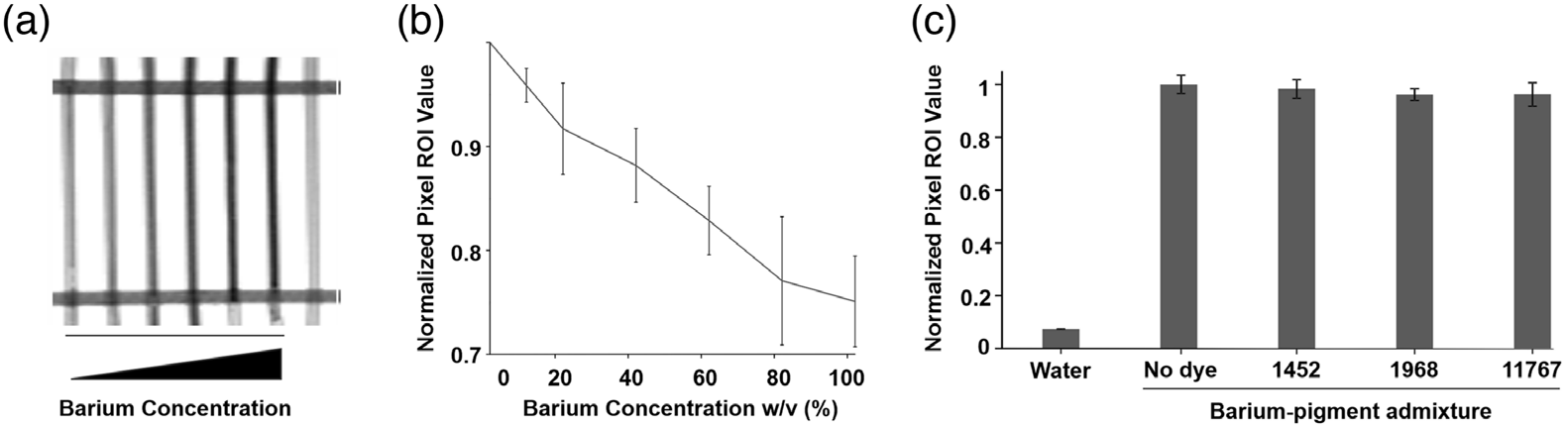
X-ray phantoms: (a) Phantom with (left to right) 0, 20, 40, 60, 80, 100% w/v barium, and water. (b) Normalized average pixel x-ray values from dye and barium. (c) Normalized average pixel x-ray values from barium/dye formulations from phantoms imaging.

After pigment screening led to the selection of 1968 for further study, x-ray images were obtained to evaluate the barium signal. The barium-pigment admixture was placed in a phantom at different concentrations to test the signal strength [Fig. 4(a)]. X-ray contrast was apparent during imaging. In Fig. 4(b), the phantom was evaluated with quantification of average pixel values, revealing a linear trend with respect to barium concentration and signal strength. Figure 4(c) shows a negligible difference between barium and the addition of dye to the phantom. The barium sulfate concentration was chosen to be 40% w/v due to the strong x-ray contrast signal as well as to match commercial orally-administered barium sulfate suspensions.

**Fig. 5:**
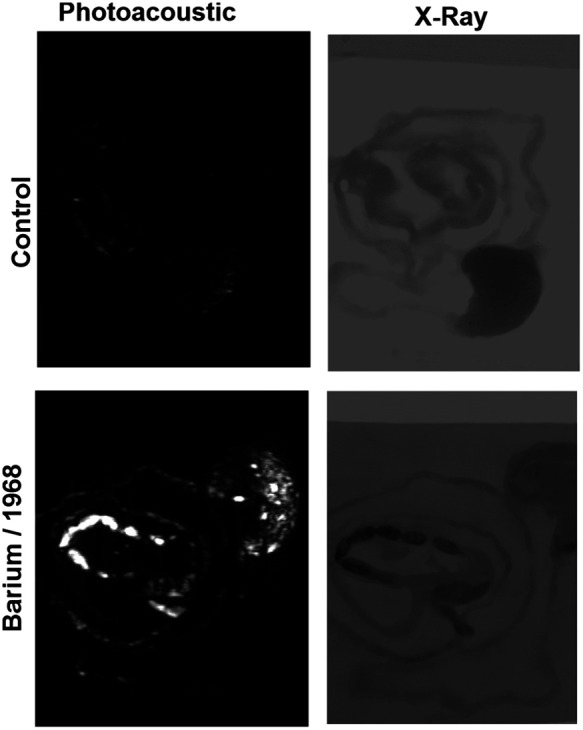
*Ex-vivo* imaging. Intestine images with PA or x-ray (left or right) with control (upper) and ingested barium-1968 contrast (lower) 4 h following oral gavage of mice.

*Ex-vivo* intestinal imaging was used to assess the barium-1968 admixture. 4 h following oral gavage, mice were sacrificed and the GI tract was excised for imaging. As shown in Fig. 5, compared to a control mouse, the PA signal was strongly observed. X-ray contrasts were also apparent within the intestines. Importantly, the x-ray contrast was generally consistent with the PA contrast with respect to anatomical localization in the intestine and stomach.

**Fig. 6:**
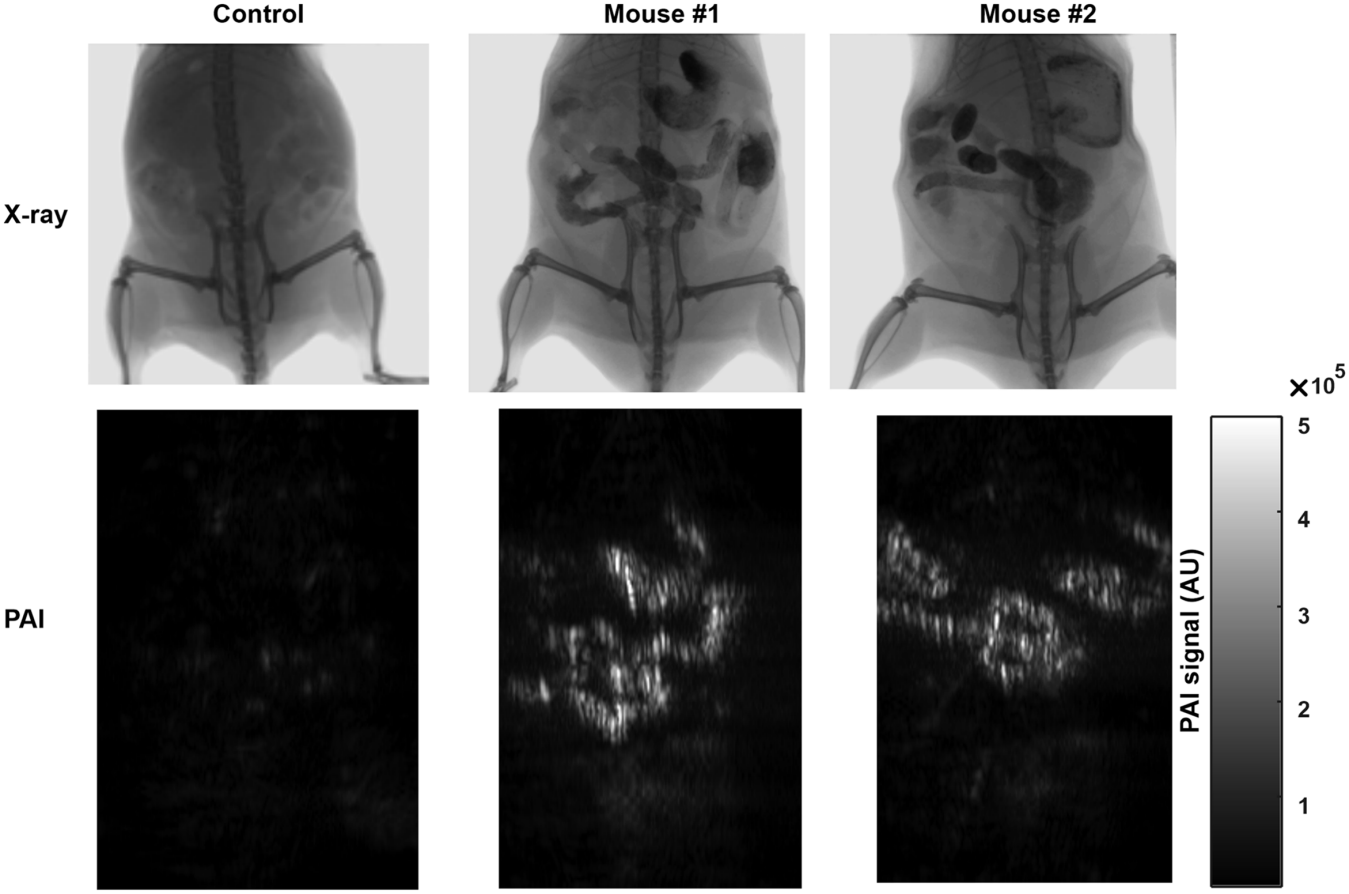
Bimodal whole body x-ray and PA imaging with barium-1968 contrast. Mice were gavaged with contrast agent and 4 h later sacrificed and subjected to whole body x-ray (upper panel) and PA (lower panel). One control mouse and two contrast-administered mice were used.

Whole-body imaging was next acquired (Fig. 6). The top row displays x-ray images of three different mice: one control mouse along with two experimental mice four hours post-gavage. Each of the mice was imaged with PAI at the same time point. There was minimal signal in the control, illustrating high PAI sensitivity to this contrast agent. The x-ray modality shows the spatial resolution of the entire mouse, including skeletal features, while PAI gives a strong signal only where the contrast agent is present.

*In-vivo* PAI was next acquired using a setup as shown in Fig. S1 in the Supplementary Materials. Mice were gavaged with barium-1968 and imaged under anesthesia 1 hour later. As shown in Fig. S2A in the Supplementary Materials, in the mice that received contrast administration, the outline of the PA contrast in the intestine was clearly visible, whereas a control mouse that did not receive the contrast agent did not show strong PA signals. Furthermore, region of interest analysis was performed to detect the movement of the contrast through the intestine. As shown in Fig. S2B in the Supplementary Materials, the rate of peristalsis was roughly 24 Hz, which is consistent with the expected values.^33^

The tolerability of barium-1968 contrast agent was assessed by weight monitoring following a single gavage dose. No differences in weight were detected in the thirteen days of monitoring post-gavage compared to an untreated control group. A complete blood count (CBC) and sera panel were assessed following the weight monitoring. After the assessment of measured parameters, no unusual differences between the treatment group and control group were observed (Table S1 in the Supplementary Materials).

## 3 Conclusion

In summary, a contrast agent comprising a commercially available NIR-II contrast agent admixed with 40% w/v barium sulfate, was easily prepared and had inherent multimodal contrast imaging properties for photoacoustic and x-ray imaging. After oral administration in mice, in-vivo imaging showed similar patterns of localization of the barium-1968 pigment in both x-ray and PAI. While a preliminary study showed this contrast agent appeared safe in mice, future studies should explore excretion kinetics of the dye. Additional future studies should also determine the utility of this dye in different animal models for functional imaging purposes.

## 4 Appendix: Experimental Materials and Methods

### 4.1 Contrast Agents

920MC and 1065A pigments were purchased from American Dye Source (catalog numbers ADS920MC and ADS1065A, respectively). 7630, 3958, 3734, and 1906 were purchased from Sands Corp H. W. with catalog numbers: SDA7630, SDA3958, SDA3734, SDA1906, respectively. 1968, 1451, 1452, 1448, 8734, 1966, and 11767 were purchased from Spectrum Dyes & Chemicals with catalog numbers S01968, S01451, S01452, S01448, S08734, S01966, and S11767, respectively. For the three dyes examined in more detail after initial screening (11767, 1452, and 1968), purity, as provided by the supplier, was greater than 98%, 95%, and 96%, respectively. The 1968 dye had extinction coefficients of $285900\;M^{-1}\;{\mathrm{cm}}^{-1}$ in methylene chloride and $221800\;M^{-1}\;{\mathrm{cm}}^{-1}$ in methanol, as reported by the supplier. Barium sulfate was purchased from Sigma Aldrich (catalog number: 7727-43-7).

### 4.2 Barium Dye Preparation and Characterization

5 mg of pigment was weighed and combined with 400 mg of barium, and water was added to bring the volume to 1 ml for a 40% w/v barium solution with 5 mg/ml of pigment. The mixture was sonicated for 30 min at room temperature. To determine the optical properties, 100  μl of prepared contrast agent was removed and then centrifuged for 3 min at 1000 g. Then from the supernatant, 10  μl was taken out without extracting any floating dye particles, and diluted into 990  μl of methanol. Absorption readings were taken at both 1064 nm and the wavelength corresponding to the maximum absorbance. These two points were multiplied by 100 for calculated supernatant absorbance. For the pellet absorption, all of the supernatant was removed and 100  μl of methanol was added. The background value from the presence of barium alone was subtracted. All absorbance measurements were carried out using a Perkin Elmer UV/Vis Lambda 365 spectrophotometer.

Wall stickiness and dye separation were quantitatively ranked on a scale from 0–2 following visual observation, with each score representing low, medium, and high, respectively. Low wall stickiness reflects that the mixture showed little to no adsorption on the interior of the glass vial. If the vial were to be inverted, the residual mixture on the glass of the vials would slide down quickly to the bottom, as aqueous solutions do. High wall stickiness was defined by a lack of movement of the mixture down the vial walls after inversion suggesting the affinity for the glass surface. The same principles apply to dye separation in which the contents of the vial are observed visually to see if the mixture is homogenous following a 30-min sonication period. If multiple large aggregates of pigment are visible, the mixture would be classified as having high dye separation.

### 4.3 Photoacoustic Imaging

Photoacoustic experiments shared the same setup reported previously,^44^ as shown in Fig. S1 in the Supplementary Materials. For the imaging experiment, all animal experiments were approved by the Institutional Animal Care and Use Committee (IACUC) at University at Buffalo under the approved animal protocols. Before imaging, mice were first overnight fasted with access to only water. The following day, mice were prepared by shaving abdominal hair, and were gavaged with 0.2 ml of the 1968/barium contrast agent with a 20-min staggered gavage schedule. This would allow time to properly image the mice at the designated time points. The 0.2-ml dosage is the maximum gavage dosage for 20 g mice as recommended by the IACUC. The mice were then anesthetized in a sealed chamber of isoflurane prior to ultrasound gel being applied to the shaved abdominal region. One hour after the gavage, the mice were placed on the imaging window, and the ultrasound imaging modality was used to locate the contrast agent in the GI tract in real time prior to capturing the photoacoustic images of the region. The staggered timing of the gavages allows for ample time to locate the contrast agent and capture the photoacoustic images at accurate time points.

The photoacoustic images capturing the dynamic intestine peristalsis used a customized transducer (Imasonics, Inc.) consisting of a 128-element linear transducer with a central frequency of 2.25 MHz and a length of 86mm. The transducer was parallel to the sagittal plane of mice with their intestines in the imaging plane of the transducer. Photoacoustic signal excitation comes from a 10-Hz pulsed Nd: YAG laser with 10 ns pulse duration and 1064 nm output. The laser output was coupled to an optical fiber bundle and redirected to the mouse. The laser output of the fiber was $24\;\mathrm{mJ}/{\mathrm{cm}}^{2}$, which is below the American National Standards Institute (ANSI) safety limitation of $100\;\mathrm{mJ}/{\mathrm{cm}}^{2}$ for the 1064-nm wavelength.^45^ The ultrasound and photoacoustic data were acquired sequentially. For photoacoustic data, the raw channel data were received and reconstructed using the back-projection algorithm in MATLAB. The ultrasound data were reconstructed using the built-in program provided by Verasonics. For peristalttic movement, after finding the approximate central to lower abdominal region, 20 photoacoustic images were captured per second. These images were then compiled by MATLAB for region of interest analysis of contrast movement.

### 4.4 Photoacoustic Microscopy

To measure the PA signals, we used a photoacoustic microscopy system, which is composed of a 1064 nm Q-switched Nd:YAG microchip laser (SNP-20F-100, Teem Photonics, France). The output laser beam was expanded and collimated by lens pair, whose focal length is 30 mm (AC254-050-AB, Thorlabs) and 75 mm (AC254-100-AB, Thorlabs), respectively. The collimated beam was focused by an objective lens (AC254-100-AB, Thorlabs) and passed to the customized opto-acoustic beam combiner, which is composed of two right-angle prisms (32-330 and 32-331, Edmund Optics), a correction lens (67-147, Edmund Optics), and an acoustic lens (45-358, Edmund Optics). The output beam from the beam combiner was reflected by a two-axis galvanometer scanner (GVS 102, Thorlabs) with the laboratory-developed waterproof method^46^ for volumetric scanning. It illuminated the sample through a window to transmit the optical beam and PA signal. To convert the generated PA signals into electrical signals, an ultrasound transducer (V214-BB-RM, Olympus NDT, Japan) was utilized and amplified by serially connected two-amplifiers (ZFL-500LN+, Mini-Circuits). The amplified analog signal was transformed into a 12-bit digital signal and recorded by the digitizer (ATS9350, Alazar Technologies, Canada).

### 4.5 X-Ray Imaging

X-ray imaging was done in a custom-built Micro-CT^47^ with a 49.5-μm pixel complementary metal-oxide semiconductor detector (Teledyne DALSA, Waterloo, Ontario). The mice were scanned using an Oxford Instruments (Oxford Instruments, Abingdon, United Kingdom) x-ray tube at a tube voltage of 70 kV, tube current of 1 mA, and thus tube energy of 70 W. Each x-ray image was flat and dark field corrected to account for non-uniformity in pixel sensitivity and detector noise, respectively.^48^

### 4.6 Toxicity Study

To determine any potential toxicity of the prepared dye admixture, a toxicity study consisting of weight monitoring was conducted. In addition, a CBC and sera biochemistry panel were conducted using two groups with n=5 mice in the control and experimental groups. Each mouse in the experimental group was orally gavaged 0.2 ml for 20 g mice. Mice were then placed in starting groups based on weight after it was determined that there was no significant difference between the means of the control and experimental groups (Fig. S3 in the Supplementary Materials). After initial weights were gathered, mice were weighed for 13 days post-gavage. At the conclusion of the weight monitoring study, a CBC test and blood sera panel were conducted. Blood samples were collected through retro-orbital bleeding of the mice, and an Element HT5 Veterinary Hematology Analyzer was used to conduct the CBC test, and an Element DC Veterinary Chemistry Analyzer was used for individual comprehensive blood chemistry panel. In total, 34 different parameters were as listed in Table S1 in the Supplementary Materials.

## References

[1] Rieffel J. Chitgupi U. Lovell J. F., “Recent advances in higher-order, multimodal, biomedical imaging agents,” Small 11, 4445–4461 (2015). SMALBC 1613-6810 10.1002/smll.201500735 26185099 PMC4582016

[2] Gao K. et al., “W-doped TiO_2_ nanoparticles with strong absorption in the NIR-II window for photoacoustic/CT dual-modal imaging and synergistic thermoradiotherapy of tumors,” Theranostics 9, 5214–5226 (2019). 10.7150/thno.33574 31410211 PMC6691582

[3] Yu X. et al., “Ultrasmall semimetal nanoparticles of bismuth for dual-modal computed tomography/photoacoustic imaging and synergistic thermoradiotherapy,” ACS Nano 11, 3990–4001 (2017). ANCAC3 1936-0851 10.1021/acsnano.7b00476 28395135

[4] Hui X. Malik M. O. A. Pramanik M., “Looking deep inside tissue with photoacoustic molecular probes: a review,” J. Biomed. Opt. 27, 070901 (2022). JBOPFO 1083-3668 10.1117/1.JBO.27.7.070901 36451698 PMC9307281

[r5] Upputuri P. K. Pramanik M., “Recent advances in photoacoustic contrast agents for in vivo imaging,” WIREs Nanomed. Nanobiotechnol. 12, e1618 (2020). 10.1002/wnan.1618 32027784

[r6] Fu Q. et al., “Photoacoustic imaging: contrast agents and their biomedical applications,” Adv. Mater. 31, 1805875 (2019). ADVMEW 0935-9648 10.1002/adma.201805875 30556205

[r7] Borg R. E. Rochford J., “Molecular photoacoustic contrast agents: design principles & applications,” Photochem. Photobiol. 94, 1175–1209 (2018). PHCBAP 0031-8655 10.1111/php.12967 29953628 PMC6252265

[r8] Han S. et al., “Contrast agents for photoacoustic imaging: a review focusing on the wavelength range,” Biosensors 12, 594 (2022). BISSED 0265-928X 10.3390/bios12080594 36004990 PMC9406114

[r9] Park E.-Y. et al., “New contrast agents for photoacoustic imaging and theranostics: recent 5-year overview on phthalocyanine/naphthalocyanine-based nanoparticles,” APL Bioeng. 5, 031510 (2021). 10.1063/5.0047660 34368604 PMC8325568

[r10] Chitgupi U. Lovell J. F., “Naphthalocyanines as contrast agents for photoacoustic and multimodal imaging,” Biomed. Eng. Lett. 8, 215–221 (2018). 10.1007/s13534-018-0059-2 30603204 PMC6208521

[11] Li M. et al., “Sound out the deep colors: photoacoustic molecular imaging at new depths,” Mol. Imaging 19, 1536012120981518 (2020). 10.1177/1536012120981518 33336621 PMC7750763

[12] Jakhmola A. Anton N. Vandamme T. F., “Inorganic nanoparticles based contrast agents for x-ray computed tomography,” Adv. Healthc. Mater. 1, 413–431 (2012). 10.1002/adhm.201200032 23184772

[r13] Aslan N. et al., “Metallic nanoparticles as X-Ray computed tomography (CT) contrast agents: a review,” J. Mol. Struct. 1219, 128599 (2020). JMOSB4 0022-2860 10.1016/j.molstruc.2020.128599

[14] Cole L. E. et al., “Gold nanoparticles as contrast agents in x-ray imaging and computed tomography,” Nanomedicine 10, 321–341 (2015).1743-5889 10.2217/nnm.14.171 25600973

[15] Sun C. et al., “*J*-aggregates of cyanine dye for NIR-II *in vivo* dynamic vascular imaging beyond 1500 nm,” J. Am. Chem. Soc. 141, 19221–19225 (2019). JACSAT 0002-7863 10.1021/jacs.9b10043 31746598

[r16] Wan H. et al., “A bright organic NIR-II nanofluorophore for three-dimensional imaging into biological tissues,” Nat. Commun. 9, 1171 (2018). NCAOBW 2041-1723 10.1038/s41467-018-03505-4 29563581 PMC5862886

[r17] Upputuri P. K. Pramanik M., “Photoacoustic imaging in the second near-infrared window: a review,” J. Biomed. Opt. 24, 040901 (2019). JBOPFO 1083-3668 10.1117/1.JBO.24.4.040901 30968648 PMC6990072

[r18] Huang K. et al., “Nanomaterials for photoacoustic imaging in the second near-infrared window,” Biomater. Sci. 7, 472–479 (2019). 10.1039/C8BM00642C 30255873

[r19] Zhang J. et al., “Development of second near-infrared photoacoustic imaging agents,” Trends Chem. 3, 305–317 (2021). 10.1016/j.trechm.2021.01.002

[r20] Ge X. et al., “Photoacoustic imaging and photothermal therapy in the second near-infrared window,” New J. Chem. 43, 8835–8851 (2019). NJCHE5 1144-0546 10.1039/C9NJ01402K

[21] Li Z. et al., “NIR-II functional materials for photoacoustic theranostics,” Bioconj. Chem. 33, 67–86 (2022). BCCHES 1043-1802 10.1021/acs.bioconjchem.1c00520 34995076

[22] Antaris A. L. et al., “A small-molecule dye for NIR-II imaging,” Nat. Mater. 15, 235–242 (2016). NMAACR 1476-1122 10.1038/nmat4476 26595119

[r23] Chitgupi U. et al., “Surfactant-stripped micelles for NIR-II photoacoustic imaging through 12 cm of breast tissue and whole human breasts,” Adv. Mater. 31, 1902279 (2019). ADVMEW 0935-9648 10.1002/adma.201902279 PMC6773519 31414515

[r24] Zhou Y. et al., “A phosphorus phthalocyanine formulation with intense absorbance at 1000 nm for deep optical imaging,” Theranostics 6, 688–697 (2016). 10.7150/thno.14555 27022416 PMC4805663

[25] Lei Z. Zhang F., “Molecular engineering of NIR-II fluorophores for improved biomedical detection,” Angew. Chem. Int. Ed. 60, 16294–16308 (2021). 10.1002/anie.202007040 32780466

[26] Kilian H. I. et al., “Facile formulation of a long-wavelength cyanine for optical imaging in the second near-infrared window,” Biomater. Sci. 8, 4199–4205 (2020). 10.1039/D0BM00572J 32515752 PMC7390685

[27] Kilian H. I. et al., “Intraperitoneal administration for sustained photoacoustic contrast agent imaging,” Photoacoustics 28, 100406 (2022). 10.1016/j.pacs.2022.100406 36213764 PMC9535324

[28] Yang X. Lovell J. F. Zhang Y., “Ingestible contrast agents for gastrointestinal imaging,” ChemBioChem 20, 462–473 (2019). CBCHFX 1439-4227 10.1002/cbic.201800589 30421487

[29] Anderson N. G. et al., “Spectroscopic (multi-energy) CT distinguishes iodine and barium contrast material in MICE,” Eur. Radiol. 20, 2126–2134 (2010). 10.1007/s00330-010-1768-9 20309554

[r30] Myagmarjalbuu B. et al., “Establishment of a protocol for determining gastrointestinal transit time in mice using barium and radiopaque markers,” Korean J. Radiol. 14, 45–50 (2013). 10.3348/kjr.2013.14.1.45 23323030 PMC3542302

[31] Kondo S., “Microinfusion method using barium sulfate for visualization of embryonic blood vessels with light and scanning electron microscopy,” J. Electron. Microsc. 45, 163–167 (1996). 10.1093/oxfordjournals.jmicro.a023427 8691091

[32] Saphier S. et al., “Gastro intestinal tracking and gastric emptying of solid dosage forms in rats using X-ray imagining,” Int. J. Pharm. 388, 190–195 (2010). IJPHDE 0378-5173 10.1016/j.ijpharm.2010.01.001 20079410

[33] Zhang Y. et al., “Non-invasive multimodal functional imaging of the intestine with frozen micellar naphthalocyanines,” Nat. Nanotechnol. 9, 631–638 (2014). NNAABX 1748-3387 10.1038/nnano.2014.130 24997526 PMC4130353

[r34] Jiang Z. et al., “Surfactant-stripped micelles with aggregation-induced enhanced emission for bimodal gut imaging in vivo and microbiota tagging ex vivo,” Adv. Healthc. Mater. 10, 2100356 (2021). 10.1002/adhm.202100356 34160147

[35] Zhang Y. et al., “Surfactant-stripped frozen pheophytin micelles for multimodal gut imaging,” Adv. Mater. 28, 8524–8530 (2016). ADVMEW 0935-9648 10.1002/adma.201602373 27396479 PMC5142297

[36] Wang D. et al., “Ingestible roasted barley for contrast-enhanced photoacoustic imaging in animal and human subjects,” Biomaterials 175, 72–81 (2018). BIMADU 0142-9612 10.1016/j.biomaterials.2018.05.016 29803105 PMC6010199

[37] Zhang E. Z. et al., “Multimodal photoacoustic and optical coherence tomography scanner using an all optical detection scheme for 3D morphological skin imaging,” Biomed. Opt. Express 2, 2202–2215 (2011). BOEICL 2156-7085 10.1364/BOE.2.002202 21833358 PMC3149519

[r38] Akers W. J. et al., “Multimodal sentinel lymph node mapping with single-photon emission computed tomography (SPECT)/computed tomography (CT) and photoacoustic tomography,” Transl. Res. 159, 175–181 (2012). 10.1016/j.trsl.2011.09.006 22340767 PMC3286037

[39] Jeon M. Kim C., “Multimodal photoacoustic tomography,” IEEE Trans. Multimedia 15, 975–982 (2013). 10.1109/TMM.2013.2244203

[40] Chen Y.-S. et al., “Dynamic contrast-enhanced photoacoustic imaging using photothermal stimuli-responsive composite nanomodulators,” Nat. Commun. 8, 15782 (2017). NCAOBW 2041-1723 10.1038/ncomms15782 28593942 PMC5472754

[41] Liao L.-D. et al., “Imaging of temperature dependent hemodynamics in the rat sciatic nerve by functional photoacoustic microscopy,” Biomed. Eng. Online 12, 120 (2013). 10.1186/1475-925X-12-120 24245952 PMC4225521

[42] Ma L. et al., “Polydopamine-coated downconversion nanoparticle as an efficient dual-modal near-infrared-II fluorescence and photoacoustic contrast agent for non-invasive visualization of gastrointestinal tract *in vivo*,” Biosens. Bioelectron. 151, 112000 (2020). BBIOE4 0956-5663 10.1016/j.bios.2019.112000 31999595 PMC7992503

[43] Xu W. Wang D. Tang B. Z., “NIR-II AIEgens: a win–win integration towards bioapplications,” Angew. Chem. Int. Ed. Engl. 60, 7476–7487 (2021). 10.1002/anie.202005899 32515530

[44] Wang Y. et al., “Optimizing the light delivery of linear-array-based photoacoustic systems by double acoustic reflectors,” Sci. Rep. 8, 13004 (2018). SRCEC3 2045-2322 10.1038/s41598-018-31430-5 30158556 PMC6115359

[45] A. N. S. Institute, American National Standard for Safe Use of Lasers, Laser Institute of America (2007).

[46] Lee J. et al., “Fully waterproof two-axis galvanometer scanner for enhanced wide-field optical-resolution photoacoustic microscopy,” Opt. Lett. 45, 865–868 (2020). OPLEDP 0146-9592 10.1364/OL.380032 32058491

[47] Ionita C. N. et al., “Cone-beam micro-CT system based on LabVIEW software,” J. Digit. Imaging 21, 296–305 (2008). JDIMEW 10.1007/s10278-007-9024-9 17333411 PMC2553273

[48] Patel V. et al., “Self-calibration of a cone-beam micro-CT system,” Med. Phys. 36, 48–58 (2009). MPHYA6 0094-2405 10.1118/1.3026615 19235373 PMC2663400

