# Supplementary material for: Barium sulfate and pigment admixture for photoacoustic and x-ray contrast imaging of the gut
Source: J Biomed Opt. 2023 Feb 10;28(8):082803. doi: 10.1117/1.JBO.28.8.082803 (PMC9917716; doi:10.1117/1.JBO.28.8.082803)
Supplement: Supplementary file 1 [file JBO_028_082803_SD001.pdf]

Supplementary Information: A Barium Sulfate-Pigment Admixture for Photoacoustic and X-ray Contrast Gut Imaging

Hailey I Kilian, Huijuan Zhang, Mohammad Mahdi Shiraz Bhurwani, Anoop M Nilam, Daewoon Seong, Mansik Jeon, Ciprian N. Ionita, Jun Xia, Jonathan F Lovell

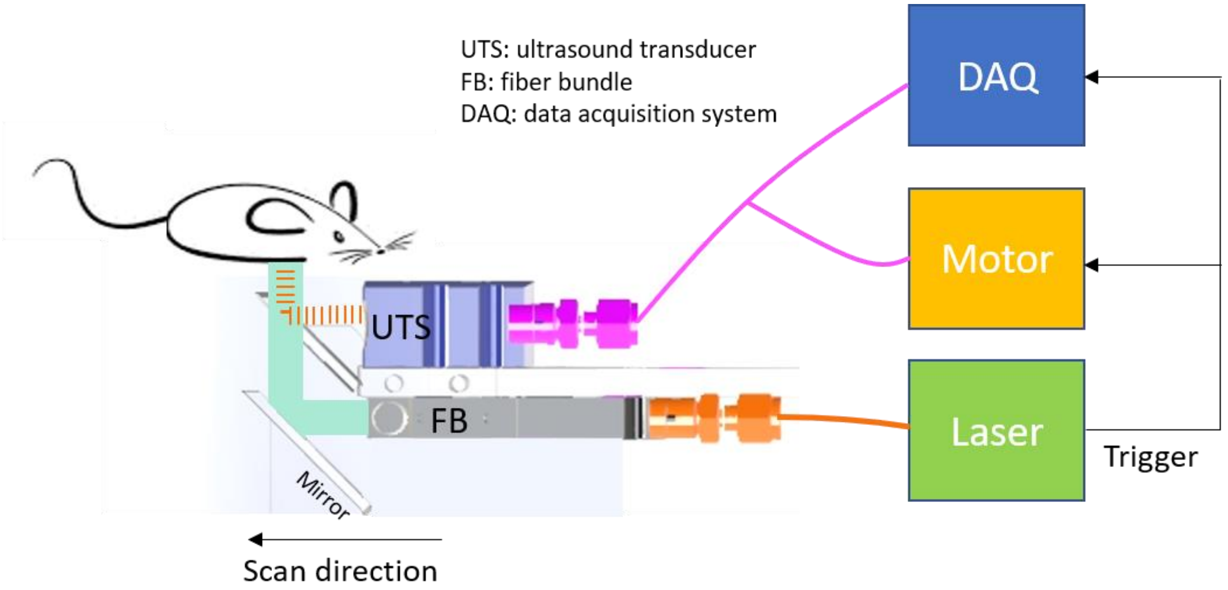

Figure S1. Schematic of setup for photoacoustic imaging.

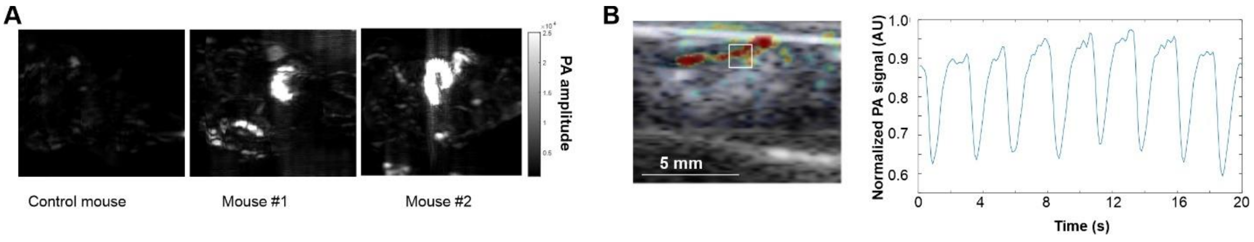

Figure S2. Functional in vivo PA imaging. Barium-1968 dye was administered to mice, and 30 min later they were imaged under anesthesia in vivo using PAI and a 1064 nm pulsed laser source. **A)** Intestinal features were visible with PAI in mice gavaged the contrast agent. **B)** Region of interest analysis demonstrating segmentation in the intestine of the contrast agent. Representative data shown from 3 biological replicates.

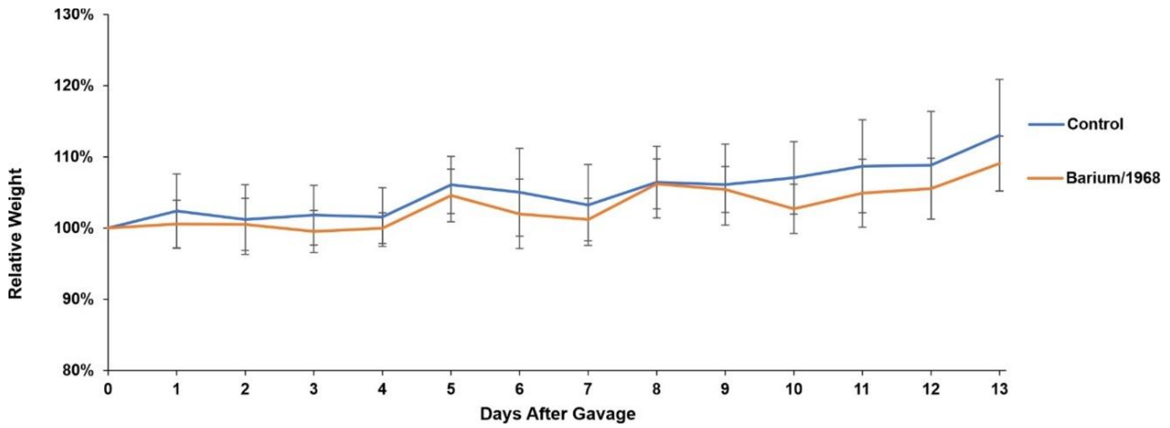

Figure S3. Impact of contrast agent administration on mouse weight. Barium-1968 pigment was administered by gavage on day 0 and mouse weight was followed (n=5 mice per group). The same dose of barium-1968 as the imaging study was used. No differences in relative mouse weight were observed by the t-test.

Table S1. CBC & Sera Panel Results: Average values in the control and experimental groups are shown for the measured parameters, listed with standard deviation. Calculated p-values are also provided for t-test.

	Control group	Gavage group	p-value
**WBC ($x10^3/\mu L$)**	7±3	6±1	0.38
**NEU ($x10^3/\mu L$)**	1±0.5	0.7±0.3	0.38
**LYM ($x10^3/\mu L$)**	5±2	5±1	0.45
**MONO ($x10^3/\mu L$)**	0.2±0.07	0.1±0.05	0.42
**EOS ($x10^3/\mu L$)**	0.3±0.2	0.1±0.08	0.16
**BAS ($x10^3/\mu L$)**	0.07±0.08	0.3±0.02	0.26
**NEU %**	10±5	10±4	0.83
**LYM %**	80±4	80±4	0.38
**MONO %**	2±0.5	2±0.6	0.91
**EOS %**	4±2	3±1	0.16
**BAS %**	0.9±0.6	0.4±0.3	0.45
**RBC ($x10^6/\mu L$)**	8.0±0.5	8.1±0.6	0.75
**HGB (g/dL)**	10±0.7	10±0.9	0.36
**HCT %**	40±2	40±3	0.33
**MCV (fL)**	50±2	50±1	0.62
**MCH (pg)**	20±0.5	20±0.4	0.17
**MCHC (g/dL)**	30±1	30±0.5	1
**RDW (%)**	10±0.7	10±0.8	0.80
**PLT ($x10^4/\mu L$)**	100±8	100±30	0.25
**MPV (fL)**	5±0.3	5±0.3	0.56
**BUN (mg/dL)**	20±6	20±5	0.16
**Phosphorous (mg/dL)**	7±3	9±1	0.21
**Calcium (mg/dL)**	5±3	8±3	0.32
**Total Protein (g/dL)**	5±0.4	10±10	0.33
**Albumin (g/dL)**	3±0.2	3±0.4	0.85
**Globulin (g/dL)**	3±0.3	3±0.2	0.38
**Albumin/Globulin Ratio**	1±0.1	1±0.1	0.53
**Glucose (mg/dL)**	170±70	200±20	0.34
**Cholesterol (mg/dL)**	200±100	200±100	0.91
**ALT (GPT)**	90±90	70±40	0.71
**ALP**	110±20	110±10	0.91
**GGT**	50±50	100±200	0.66
**Total Bilirubin**	0.6±0.6	1±1	0.40
